# Interaction between SCP3 and JAB1 Confers Cancer Therapeutic Resistance and Stem-like Properties through EGF Expression

**DOI:** 10.3390/ijms22168839

**Published:** 2021-08-17

**Authors:** Se Jin Oh, Kyung Hee Noh, Kwon-Ho Song, Tae Woo Kim

**Affiliations:** 1 BK21 Graduate Program, Department of Biomedical Sciences, Korea University College of Medicine, Seoul 02841, Korea; ziziana87@korea.ac.kr; 2 Department of Biochemistry and Molecular Biology, Korea University College of Medicine, Seoul 02841, Korea; 3 Gene Therapy Research Unit, Korea Research Institute of Bioscience and Biotechnology, Daejeon 34141, Korea; trollius@kribb.re.kr; 4 Department of Cell Biology, Daegu Catholic University School of Medicine, Daegu 42472, Korea

**Keywords:** synaptonemal complex protein 3 (SCP3), Jun activation domain-binding protein 1 (JAB1), epidermal growth factor (EGF), epidermal growth factor receptor (EGFR), AKT, cancer, immune resistance, chemo-resistance, cancer stem cell (CSC)

## Abstract

Synaptonemal complex protein 3 (SCP3), a member of the Cor1 family, has been implicated in cancer progression, and therapeutic resistance, as well as cancer stem cell (CSC)-like properties. Previously, we demonstrated that SCP3 promotes these aggressive phenotypes via hyperactivation of the AKT signaling pathway; however, the underlying mechanisms responsible for SCP3-induced AKT activation remain to be elucidated. In this study, we demonstrated that the EGF-EGFR axis is the primary route through which SCP3 acts to activate AKT signaling. SCP3 triggers the EGFR-AKT pathway through transcriptional activation of EGF. Notably, neutralization of secreted EGF by its specific monoclonal antibody reversed SCP3-mediated aggressive phenotypes with a concomitant reversal of EGFR-AKT activation. In an effort to elucidate the molecular mechanisms underlying SCP3-induced transcriptional activation of EGF, we identified Jun activation domain-binding protein 1 (JAB1) as a binding partner of SCP3 using a yeast two-hybrid (Y2H) assay system, and we demonstrated that SCP3 induces EGF transcription through physical interaction with JAB1. Thus, our findings establish a firm molecular link among SCP3, EGFR, and AKT by identifying the novel roles of SCP3 in transcriptional regulation. We believe that these findings hold important implications for controlling SCP3^high^ therapeutic-refractory cancer.

## 1. Introduction

Synaptonemal complex protein 3 (SCP3), a member of the Cor1 family, is a structural component of the synaptonemal complex, which mediates synapsis, pairing of homologous chromosomes during meiosis in germ cells [1]; hence, it is mainly expressed in the testis and ovary [2]. However, overexpression of SCP3 is frequently observed in various types of cancers, including acute lymphoblastic leukemia, non-small cell lung cancer and cervical cancer [3,4,5]. Furthermore, previous studies provide evidence that SCP3 is important for tumor progression, metastasis, and immune resistance, as well as cancer stem cell (CSC)-like property [6,7,8]. In this regard, we demonstrated a pathway involved in SCP3-mediated multi-aggressive phenotypes of tumor cells, which is dependent on AKT signaling [6,8]. However, the underlying mechanisms responsible for SCP3-induced AKT activation remains to be elucidated.

Jun activation domain-binding protein-1 (JAB1) has previously been described as a coactivator of the AP1 transcription factor [9] and is evolutionarily conserved in humans, mice, fission yeast, and plants, which provides evidence that JAB1 is critical to cell survival and proliferation [10,11,12]. Indeed, accumulating evidence demonstrated that JAB1 was overexpressed and usually associated with poor prognosis in a variety of human malignancies [13,14]. In this regard, JAB1 is involved in multiple protein interactions that affect many stages of tumorigenesis, and therefore, it has the potential to be an effective therapeutic target [15]. However, the functional association between JAB1 and SCP3 remains largely unknown.

In this study, to gain an in-depth understanding of the molecular mechanisms by which SCP3 promotes multi-aggressive phenotypes, we focused on the precise mechanisms responsible for SCP3-induced AKT activation. In doing so, we demonstrated that SCP3 leads to transcriptional induction of EGF through physical interaction with JAB1, which contributes to subsequent activation of the EGFR-AKT signaling pathway. Thus, our findings provide insights into the novel role of SCP3 in transcriptional regulation. Furthermore, understanding of a firm molecular link between SCP3 and JAB1 may provide a rational for a new therapeutic target in controlling SCP3^+^ refractory cancer.

## 2. Results

### 2.1. SCP3 Activates AKT via EGFR Signaling

**Figure 1:**
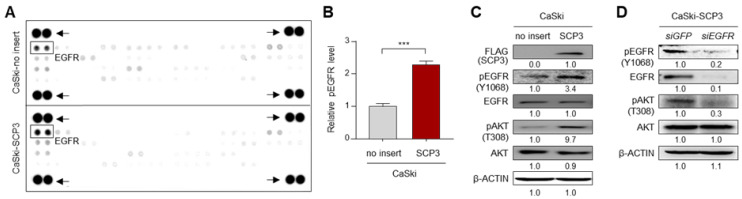
EGFR is an upstream signaling pathway responsible for SCP3-induced AKT activation. (**A**,**B**) The phosphorylation status of RTKs in CaSki-no insert and CaSki-SCP3 cells was assessed by Human phospho-RTK array. (**A**) Representative dot images from the phospho-RTK array. Boxes indicate the spots corresponding to the levels of phosphorylated EGFR. Arrows indicate the reference spots on the array blot, as a loading control. (**B**) The graph represents quantification for relative level of pEGFR on the array blot. The data represent the mean ± SD. *** *p* ≤ 0.001, by unpaired, two-tailed Student’s *t*-test. (**C**) The levels of pEGFR, EGFR, pAKT and AKT in CaSki- no insert and CaSki-SCP3 cells were confirmed by Western blots. (**D**) CaSki-SCP3 cells were transfected with *siGFP* or *siEGFR*. Levels of pEGFR, EGFR, pAKT and AKT were analyzed by Western blots. (**C**,**D**) β-ACTIN was used as an internal loading control. Numbers below blot images indicate fold-change in protein level. All experiments were performed in triplicate.

Previously, we demonstrated that SCP3 promotes immune-resistant and CSC-like phenotypes in tumor cells [6,8]. Although it is certain that activation of AKT signaling is a critical determinant for the phenotypes mediated by SCP3 [8], the underlying mechanisms responsible for SCP3-induced AKT activation are still unknown. As multiple receptor tyrosine kinases (RTKs) can mediates AKT signaling in cancer cells, a phospho-RTK antibody array was performed to identify an upstream RTK, which might have been involved in SCP3-mediated AKT activation [16]. From this analysis, we noted that the phosphorylation level of EGFR (pEGFR) was up-regulated in CaSki-SCP3 cells compared to CaSki-no insert cells (Figure 1A,B). Consistently, the CaSki-SCP3 cells showed increased levels of pEGFR and pAKT compared with CaSki-no insert cells (Figure 1C). In line with this observation, knockdown of EGFR in CaSki-SCP3 cells robustly dampened levels of pAKT (Figure 1D), indicating a crucial role of EGFR signaling in SCP3-induced AKT activation. Thus, we conclude that EGFR activation is a key upstream signaling pathway for AKT activation mediated by SCP3.

### 2.2. SCP3 Triggers EGFR Signaling through Transcriptional Activation of EGF

**Figure 2:**
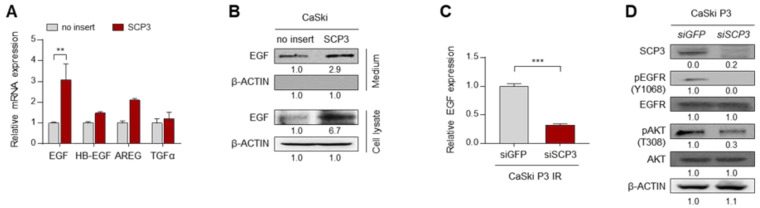
SCP3 activates EGFR-AKT signaling through transcriptional induction of EGF. (**A**) mRNA expressions of EGF, HB-EGF, AREG, and TGFα was analyzed by qRT-PCR. (**B**) The protein levels of secreted EGF and internal EGF were evaluated by Western blot analysis of the conditioned medium and lysate of these cells. (**C**,**D**) CaSki P3 cells were transfected with *siGFP* or *siSCP3*. (**C**) EGF mRNA expression was analyzed by qRT-PCR. (**D**) The levels of SCP3, pEGFR, EGFR, pAKT, and AKT were analyzed by Western blots. β-ACTIN was used as an internal loading control. (**B**,**D**). All experiments were performed in triplicate. The *p*-values were determined by unpaired, two-tailed Student’s *t*-test. The data represent the mean ± SD (** *p* ≤ 0.01, *** *p* ≤ 0.001).

Since activation of the EGFR signaling pathway is mediated by EGF and its related ligands [17], we hypothesized that SCP3-induced EGFR activation might be due to up-regulation of EGFR ligands. Intriguingly, we observed that expression of EGF gene was significantly up-regulated upon SCP3 overexpression (Figure 2A). Consistently, we also found that CaSki-SCP3 cells had markedly higher levels of both secreted and intracellular EGF proteins, compared with CaSki-no insert cells (Figure 2B). Previously, we established a highly immune-resistant cervical tumor cell line, CaSki P3, generated from its immune susceptible parental cell line, CaSki P0, through three rounds of selection by cognate cytotoxic T lymphocytes (CTLs) [18]. These immune-resistant CaSki-P3 cells exhibited up-regulated levels of SCP3 as well as hyperactivation of EGFR-AKT signaling [8]. Notably, knockdown of SCP3 in CaSki P3 cells markedly reduced the level of EGF mRNA (Figure 2C). This was accompanied by a loss of signaling of EGFR and AKT (Figure 2D). Taken together, these results suggest that SCP3 triggers EGFR signaling through transcriptional induction of EGF, therefore potentiating AKT activation.

### 2.3. Neutralization of Secreted EGF by Its Specific Monoclonal Antibody Reverses SCP3-Mediated Aggressive Phenotypes

**Figure 3:**
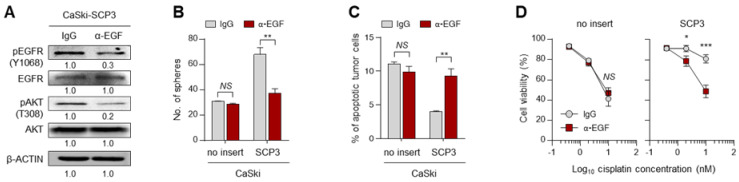
The EGF-EGFR axis plays a crucial role in SCP3-mediated multi-aggressive phenotypes. (**A**–**D**) CaSki-no insert or CaSki-SCP3 cells were treated with IgG or EGF antibody. (**A**) Western blot analysis of pEGFR, EGFR, pAKT, and AKT levels. β-ACTIN was used as an internal loading control. (**B**) Sphere-forming capacity of indicated cells. (**C**) Flow cytometry analysis of active caspase-3^+^ cells in the cells after intracellular delivery of granzyme B. (**D**) Cell viability was measured by live cell counting using trypan blue. All experiments were performed in triplicate. The *p*-values were determined by unpaired, two-tailed Student’s t-test. The data represent the mean ± SD (* *p* ≤ 0.05, ** *p* ≤ 0.01, *** *p* ≤ 0.001. *NS*, not significant).

To validate the significance of EGF in SCP3-mediated AKT activation, we neutralized EGF in the culture media of SCP3-overexpressing cells with its specific monoclonal antibody. Notably, neutralization of secreted EGF led to a significant decrease in the phosphorylation levels of EGFR and AKT in CaSki-SCP3 cells, indicating a direct role of EGF in the SCP3-induced EGFR-AKT signaling pathway (Figure 3A). Based on our previous finding that SCP3 promotes stem-like property and multi-modal resistance against to immune- and chemo-therapy [8], we wondered if EGF is required for promoting multi-aggressive phenotypes, which are mediated by SCP3. Consistent with our previous results [8], CaSki-SCP3 cells showed more sphere-forming capacity when cells were cultured under suspension conditions (Figure 3B) and they were more resistant to granzyme B-mediated apoptosis and cisplatin treatment, compared with CaSki-no cells (Figure 3C,D). Notably, blockade of the EGF-EGFR axis with an EGF antibody reduced sphere-forming capacity (Figure 3B), and it also increased the susceptibility to granzyme B and cisplatin (Figure 3C,D). In contrast with CaSki-SCP3 cells, CaSki-no cells treated with an EGF antibody did not show significantly alteration of sphere-forming capacity and the susceptibility to CTLs and cisplatin (Figure 3B–D). Thus, our findings demonstrate that the EGF-EGFR axis plays a crucial role in AKT activation as well as in various aggressive phenotypes mediated by SCP3.

### 2.4. SCP3 Physically Interacts with JAB1

**Figure 4:**
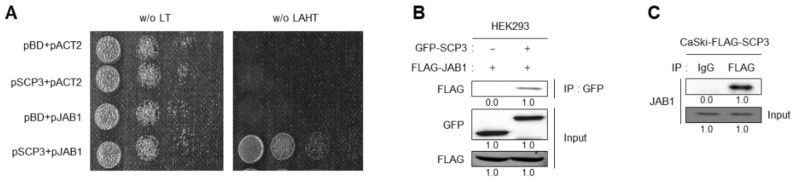
SCP3 interacts with JAB1. (**A**) Positive protein-protein interactions were determined by monitoring cell growth over 3 days on a medium lacking Leu and Trp (w/o LT), or a medium lacking Leu, Trp, His and Ade (w/o LAHT). (**B**) Co-immunoprecipitation of SCP3 and JAB1. HEK293 cells were co-transfected with the indicated constructs. Lysates of the transfected cells immunoprecipitated with the GFP antibody, followed by Western blotting using GFP and FLAG antibodies. (**C**) SCP3 bound to endogenous JAB1 in CaSki-SCP3 cells. Immunoprecipitation was performed using rabbit IgG or FLAG antibody, followed by Western blotting using the indicated antibodies. All experiments were performed in triplicate.

Although it is certain that transcriptional induction of the EGF gene is a critical determinant of SCP3-mediated AKT activation for promoting multiple aggressive phenotypes, the underlying mechanism behind how SCP3 regulates EGF transcription is largely unknown. To gain an insight into the role of SCP3 in transcriptional regulation, we employed a yeast two-hybrid system (Y2H) and screened JAB1 as a potential SCP3-interacting protein. Indeed, positive interaction of SCP3 with JAB1 was confirmed by cell growth on selective media lacking leucine (Leu), tryptophan (Trp), histidine (His), and adenine (Ade) (Figure 4A). To confirm our Y2H results, we carried out co-immunoprecipitation (co-IP) using lysates of HEK293 cells transfected with GFP-SCP3 and FLAG-JAB1. Under the IP condition by the GFP antibody, FLAG-JAB1 successfully co-precipitated with GFP-SCP3 (Figure 4B). Consistent with this finding, endogenous JAB1 could indeed co-precipitate with FLAG-SCP3 in CaSki-SCP3 cells (Figure 4C). These results indicate that SCP3 physically interacts with JAB1.

### 2.5. SCP3 Induces EGF Transcription through Physical Interaction with JAB1

**Figure 5:**
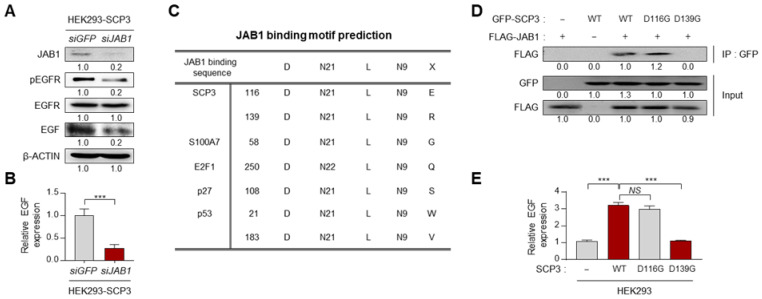
SCP3 transcriptionally up-regulates EGF through physical interaction with JAB1. (**A**,**B**) HEK293-SCP3 cells were treated with *siGFP* or *siJAB1*. (**A**) Western blot analysis of JAB1, pEGFR, EGFR, and EGF expression. β-ACTIN was used as an internal loading control. (**B**) EGF mRNA expression was analyzed by qRT-PCR. (**C**) JAB1 binding motif prediction on SCP3, S100A7, E2F1, p27, and p53. (**D**,**E**) HEK293 cells were co-transfected with the indicated constructs. (**D**) Co-immunoprecipitation of SCP3 and JAB1. Lysates of the transfected cells immunoprecipitated with GFP antibody, followed by Western blotting using GFP and FLAG antibodies. (**E**) EGF mRNA expression was analyzed by qRT-PCR. All experiments were performed in triplicate. The *p*-values were determined by unpaired, two-tailed Student’s *t*-test. The data represent the mean ± SD (*** *p* ≤ 0.001. *NS*, not significant).

We next examined whether JAB1 is required for the EGF-EGFR axis mediated by SCP3. Knockdown of JAB1 in HEK293-SCP3 cells markedly dampened the level of EGFR phosphorylation (Figure 5A), which was accompanied by decreased level of EGF protein or mRNA (Figure 5A,B). The result suggests the crucial role of JAB1 in a function of SCP3 regarding transcriptional regulation. As several amino acids are conserved within the JAB1 binding domain from different proteins and they play an essential role in interaction with JAB1 [19], we aligned the amino acid sequences of putative JAB1 binding domain in SCP3. From this analysis, we noted two putative JAB1 binding domains, D116-L138-E148 and D139-L160-E170, within the c-terminal region of SCP3 protein (Figure 5C). To examine whether the putative JAB1 binding domains within SCP3 protein are crucial for the interaction with JAB1 protein, the amino acids D116 or D139 of SCP3 protein were replaced with glycine to generate the mutant D116G and D139G, respectively (Figure 5D). Interestingly, SCP3 WT or D116G successfully bound to JAB1, whereas the SCP3 D139G failed to bind with JAB1 (Figure 5D), indicating that the D139 residue of the SCP3 protein is important for the interaction with JAB1 protein. Given the crucial role of the JAB1 in transcriptional regulation, we reasoned that SCP3-induced transcriptional activation of the EGF gene is mediated by the interaction with JAB1. Indeed, SCP3 WT or D116G led to a marked increase in EGF expression; however, SCP3 D139G failed to cause a change in the expression of EGF, compared with control (Figure 5E). Taken together, these findings suggest that SCP3 up-regulates the EGF gene through physical interaction with JAB1.

## 3. Discussion

Harnessing the immune system to detect and eliminate tumor cells, cancer immunotherapy has emerged as a promising cancer treatment [20]. However, the presence of immune-resistant tumor cells limits its clinical success. Accumulating evidence indicates that cancer immunoediting drives the adaptation of tumor cells to host immune surveillance, therefore contributing to generation of cancer cells with better survival advantages [21,22,23,24,25,26,27]. In this regard, our previous studies demonstrated that SCP3^+^ tumor cells enriched by immune selection confer preferential activation of AKT signaling, therefore promoting multiple aggressive phenotypes, such as immune-resistant, chemo-resistant, and stem-like properties [6,8]. Although we demonstrated that AKT signaling is a central channel in SCP3-mediated multiple aggressive properties, it is unclear how SCP3 activates AKT signaling. In this study, we found that the EGF-EGFR axis is the primary route through which SCP3 acts to activate the AKT signaling. Furthermore, our findings provide an insight into the novel role of SCP3 in a transcriptional regulation through physically interacting with JAB1.

EGFR signaling, one of the most dysregulated pathways in various cancers, is highly associated with tumor progression, chemoresistance, and metastasis through its downstream signaling pathway, such as PI3K/AKT signaling [28]. Here, we have elucidated that EGFR signaling is the primary route through which SCP acts to activate AKT signaling and then to promote various aggressive phenotypes, including immune-resistant, chemo-resistant, and stem-like properties. Indeed, blockade of EGF signaling with EGF neutralizing antibodies led to decrease in AKT signaling, as well as in aggressive phenotypes of SCP3-overexpressing cells. Currently, several chemical inhibitors against EGFR were developed, considering the important biological impact of EGF and their receptors in tumor cells [29]. As EGF-EGFR signaling has been implicated as a central channel in the development of multi-aggressive phenotypes by SCP3, we believe that inhibition of EGFR signaling may be an effective strategy to control refractory cancer, especially if there is high SCP3 expression.

It is certain that induction of EGF expression is critical for triggering the EGFR-AKT signaling pathway in SCP3-mediated aggressive phenotypes. In the course of elucidating how SCP3 up-regulates the EGF gene, we identified JAB1 as a physical interactor for SCP3 by Y2H, and we found that SCP3 function responsible for the transcriptional induction of the EGF gene is closely related to the JAB1. Notably, the interaction with JAB1 is essential for SCP3-induced transcriptional activation of EGF, because the SCP3 D139G mutant, which can no longer bind to JAB1, failed to increase the EGF expression. Although it will be important to assess the precise underlying mechanisms by which SCP3 regulates EGF expression in future studies, our findings provide novel insights into the role of SCP3 in transcriptional regulation. Furthermore, blockade of interaction between SCP3 and JAB may be a promising therapeutic approach for SCP3^+^ refractory cancer. However, pharmacologic inhibitors of SCP3 or JAB1 are yet to be developed. Therefore, further studies to identify agents that block the interaction between SCP3 and JAB1 are required to evaluate these possibilities.

In conclusion, we demonstrated that SCP3 confers AKT-mediated multi-aggressive phenotypes by up-regulating EGF expression. Critically, these multi-aggressive phenotypes mediated by SCP3 are dependent on EGF-EGFR signaling, which is triggered by JAB1-dependent EGF expression. Furthermore, we uncovered that the physical interaction between SCP3 and JAB1 is critical for transcriptional activation of EGF. Finally, understanding of a molecular link between SCP3 and JAB1 may provide a rationale for a new therapeutic target in controlling refractory cancer.

## 4. Materials and Methods

### 4.1. Cell Lines Reagents

CaSki and HEK293 cell lines were purchased from American Type Culture Collection (ATCC, Manassas, VA, USA) and tested for mycoplasma using Mycoplasma Detection Kit (Thermo Fisher Scientific, San Jose, CA, USA). The identities of cell lines were confirmed by short tandem repeat (STR) profiling by IDEXX Laboratories Inc. and were used within 6 months for testing. Generation and maintenance of the immune-edited CaSki P3 [18] and CaSki-SCP3 [8] cell lines were previously described. All cells were grown at 37 °C in a 5% CO_2_ incubator/humidified chamber. Cisplatin were purchased from Selleckchem (Selleckchem, Houston, TX, USA).

### 4.2. DNA Constructs

To generate p3xFLAG CMV7.1-SCP3 and pEGFP-SCP3, cDNA encoding human SCP3 were amplified from pMSCV-SCP3 construct [8], and then it was cloned into the p3xFLAG CMV7.1 (Sigma-Aldrich, St. Louis, MO, USA) and pEGFP-C1 (Clontech, Mountain View, CA, USA), respectively. FLAG-JAB1 constructs were generated with a PCR-based strategy from a human cDNA library (Clontech, Mountain View, CA, USA) with the following primers: JAB1 forward, 5′-GAATTCCATGGCGGCGTCCGGGAGC-3′; reverse 5′-GGATTCGTTAAGAGATGTTAATTTG-3′. The amplified cDNA was cloned into *Eco*RI/*Bam*HI restriction sites of the p3xFLAG CMV7.1 vector. In all cases, plasmid integrity was confirmed by DNA sequencing.

### 4.3. Site-Directed Mutagenesis

Site-directed mutagenesis was performed using a QuickChange XL Site-Directed Mutagenesis Kit (Strategene, Bellingham, WA, USA) according to the manufacturer’s instructions. For the generation of the SCP3-D116G or D139G construct the following primer set was used: D116G, 5′-GGAAAACACAACAAGGTCAAAGGCAGAAGC-3′ (forward) and 5′-GCTTCTGCCTTTGACCTTGTTGTGTTTTCC-3′ (reverse) and D139G, 5′-GCAGTGGGATTTAGGTATGCAGAAAGCTGA-3′ (forward) and 5′-TCAGCTTTCTGCATACCTAAATCCCACTGC-3′ (reverse). Thermal cycling conditions for PCR were 95 °C for 5 min; 18 cycles of 95 °C for 1 min, and 64 °C for 1 min, and 68 °C for 15 min. PCR products were digested with *Dpn*I at 37 °C for 1 h and transformed into XL10-Gold ultracompetent bacterial cells. Mutations were confirmed by DNA sequencing.

### 4.4. siRNA Constructs

Synthetic small interfering RNAs (siRNAs) specific for GFP, SCP3, and JAB1 were purchased from Bioneer (Daejeon, Korea); Non-specific GFP (green fluorescent protein), 5′-GCAUCAAGGUGAACUUCAA-3′ (sense), 5′-UUGAAGUUCACCUUGAUGC-3′ (antisense); SCP3, 5′-CAGUUAUAUGAGCAGUUCAUAAA-3′ (sense), 5′-UUUAUGUUCUGCUCAUAUAACUG-3′ (antisense); JAB1, 5′-CCAGACTATTCCACTTAATTT-3′ (sense), 5′-ATTAAGTGGAATAGTCTGGTT-3′ (antisense). siRNA was transfected in vitro into 6-well plates at a dose of 200 pmol per well using Lipofectamine 2000 (Invitrogen, Carlsbad, CA, USA) according to the manufacturer’s instructions.

### 4.5. Antibody Arrays of Phospho-RTK

Phospho-RTK arrays were performed according to the manufacturer’s recommendations (Human phospho-RTK Array Kit, R&D Systems, Minneapolis, MN, USA). Briefly, phospho-RTK array membranes were blocked with 5% BSA in TBS (0.01 M Tris-HCl, pH 7.6) for 1 h. Then, the membranes were incubated with 200 μg of total protein for 16 h at 4 °C. After extensive washing with TBS, the membranes were incubated with phospho-tyrosine HRP antibody for 2 h at room temperature. The unbound HRP antibody was washed out with TBS containing 0.1% Tween-20. Finally, each array membrane was developed using the chemiluminescence ECL detection system (Elpis Biotech, Daejeon, Korea), and signals were detected using a luminescent image analyzer (LAS-4000 Mini, Fujifilm, Tokyo, Japan).

### 4.6. Real-Time Quantitative RT-PCR

Total RNA was isolated using RNeasy Micro kit (Qiagen, Valencia, CA, USA), and complementary DNAs were synthesized by reverse transcriptase (RT) using iScript cDNA synthesis kit (Bio-Rad, Hercules, CA, USA), according to the manufacturer’s recommended protocol. Real-time quantitative PCR was performed using iQ SYBR Green super mix (Bio-Rad) with the following specific primers: EGF, 5′-GGATGGGATGTCCTCTTGCC-3′ (forward) and 5′-ACCCAGGAGCCAGGGATAA-3′ (reverse); HB-EGF, 5′-TTCTGGCTGCAGTTCTCTCG-3′ (forward) and 5′-AAGTCACGGACTTTCCGGTC-3′ (reverse); AREG, 5′-GTCGCTCTTGATACTCGGCT-3′ (forward) and 5′-CACAGGGGAAATCTCACTCCC-3′ (reverse); TGFα, 5′-CCTGTTCGCTCTGGGTATTGT-3′ (forward) and 5′-GTGGGAATCTGGGCAGTCAT-3′ (reverse); β-ACTIN, 5′-CGACAGGATGCAGAAGGAGA-3′ (forward) and 5′-TAGAAGCATTTGCGGTGGAC-3′ (reverse) on a CFX96 real-time PCR detection system. All real-time quantitative PCR experiments were performed in triplicate and quantification cycle (Cq) values were determined using Bio-Rad CFX96 Manager 3.0 software. Relative quantification of the mRNA levels was performed using the comparative Ct method with β-actin as the reference gene.

### 4.7. Tumor Sphere-Forming Assay

Cells were plated at 1 × 10^3^ cells/well in 6-well, super-low adherence vessels (Corning, New York, NY, USA) containing serum-free DMEM-F12 (Thermo Fisher Scientific, San Jose, CA, USA) supplemented with 20 ng/mL of EGF (Gibco), 20 ng/mL of basic FGF (Gibco), and 1× B27 (Gibco). The medium was replaced every three days to replenish nutrients. Colonies more than 50 um in diameter were counted under a microscope.

### 4.8. Granzyme B-Mediated Apoptosis Assay

Recombinant human granzyme B (Enzo Life Sciences, New York, NY, USA) was mixed with BioPORTER QuikEase Protein Delivery Kit (Sigma-Aldrich, St. Louis, MO, USA). 1 × 10^5^ cells per well were plated in 12-well plates and cultured overnight at 37 °C. Cells were washed, and 100 ng of granzyme B in Opti-MEM (Thermo Fisher Scientific) was added to each well. After incubation for 4 h at 37 °C, the frequency of apoptotic cells was determined by staining with active caspase-3 antibody (BD Biosciences) and examined by flow cytometry as shown gating strategy in Supplementary Figure S1.

### 4.9. Trypan Blue Exclusion Assay

For determining cell viability, trypan blue exclusion assay was performed. Briefly, cells were seeded at 1 × 10^5^ cells/well in 12-well plates 1 day prior to the assay. Cisplatin treatment was performed at the concentrations indicated in figures. After 24 h, cells were detached and stained with 0.4% trypan blue. Unstained cells were counted using a hemocytometer. Data are expressed as percentages of unstained cells compared with control cells not exposed to chemical reagents.

### 4.10. Yeast Two-Hybrid (Y2H) Assay

For bait construction with human SCP3, cDNA encoding full-length human SCP3 was cloned into the *EcoR I* and *Sal I* restriction enzyme sites of the pBD-GAL4 Cam vector (Strategene). The bait pBD-GAL4-SCP3 plasmid was transformed into a yeast strain AH109. After mating with a pre-transformation HeLa cell library (Clontech), positive clones were selected on triple-dropout plates (SD-Leu-Trp-His) and then further isolated using high stringency selectable plates (SD-Leu-Trp-His-Ade). Growth selection were confirmed using β-galactosidase activity assay and obtaining positive colonies, the purified plasmids were sequenced. A homology search in the GenBank using the BLAST program revealed that the plasmids encoded JAB1 (accession number: NM_006837). The human JAB1 cDNA encoding a full-length gene was cloned with *EcoR I* and *Xho* *I* restriction enzyme sites of the prey pAD-GAL4 plasmid vector (Strategene). pAD-JAB1 plasmid was transformed into yeast competent cells that already contained pBD-SCP3, while the transformants were selected based on their tryptophan prototrophy (plasmid vector marker) on a synthetic medium (Ura, His, Trp) containing 2% (*w*/*v*) glucose. Positive protein–protein interactions were monitored as the formation of blue colonies on the X-gal-containing medium as described previously [30].

### 4.11. Immunoprecipitation

For co-immunoprecipitation of SCP3 and JAB1, HEK293 cells expressing the indicated constructs were cultured for 48 h, and whole-cell lysates were prepared with NP40 lysis buffer (50 mM Tris-HCL, pH 8.0, 5 mM EDTA, 150 mM NaCl, 1% NP40, 1 mM PMSF) containing a protease inhibitor. Immunoprecipitation was carried out by incubation with 1 μg of GFP (598, MBL, Nagoya, Japan), FLAG (M185-3L, MBL) antibody or rabbit IgG for 16 h. For immunoprecipitation of endogenous JAB1, CaSki SCP3 cells were lysed in NP40 lysis buffer containing a protease inhibitor. Immunoprecipitation was carried out by incubation with 1 μg of anti-FLAG or rabbit IgG for 16 h. The bound proteins were eluted by boiling in SDS sample buffer and were detected by Western blotting.

### 4.12. Statistical Analysis

All data are representative of at least three separate experiments. Individual data points were compared by two-tailed Student *t*-test. In all cases, results with two-tailed *p*-values of <0.05 were considered statistically significant.

## Data Availability

Not applicable.

## Supplementary Materials

The following are available online at https://www.mdpi.com/article/10.3390/ijms22168839/s1.
