# Supplementary material for: Interaction between SCP3 and JAB1 Confers Cancer Therapeutic Resistance and Stem-like Properties through EGF Expression
Source: Int J Mol Sci. 2021 Aug 17;22(16):8839. doi: 10.3390/ijms22168839 (PMC8396186; doi:10.3390/ijms22168839)
Supplement: Supplementary file 1 [file ijms-22-08839-s001.zip › ijms-1309361-SI.pdf]

Supplementary information for the manuscript entitled:

Interaction between SCP3 and JAB1 confers cancer therapeutic resistance and stem-like properties through EGF expression

Se Jin Oh et al.

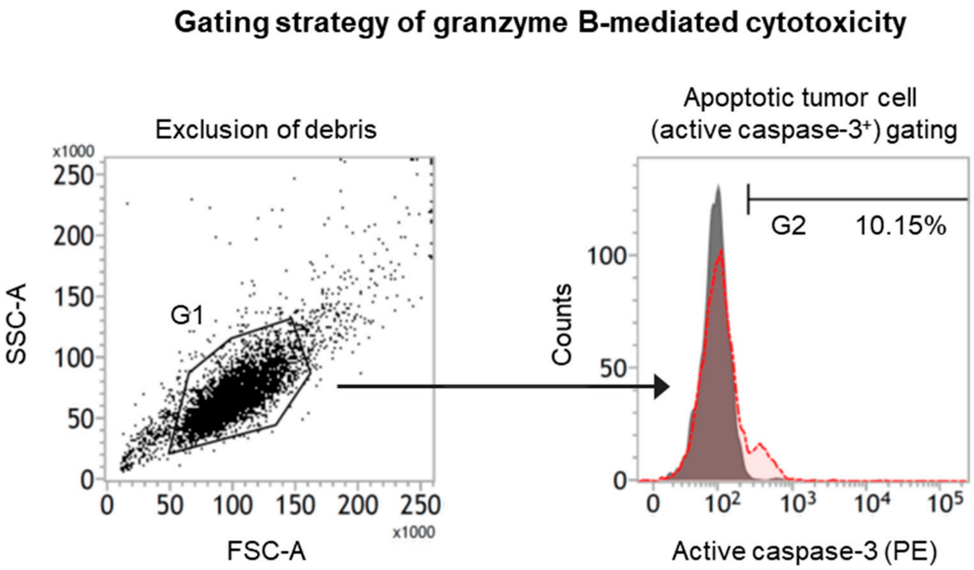

Supplementary Figure S1 Gating strategy for identification of apoptotic tumor cells by granzyme B-mediated killing. Representative flow cytometry analysis showing the gating strategy. Population was initially gated on the basis of the forward side scatter characteristics and debris were eliminated. Finally, the active caspase-3^{+} population was measured in the active caspase-3 (PE) histogram.
